# First case of fungemia caused by a rare and pan-echinocandin resistant yeast *Sporopachydermia lactativora* in China

**DOI:** 10.1080/21501203.2024.2418111

**Published:** 2024-10-28

**Authors:** Qiushi Zheng, Shuzhen Xiao, Lingyu Ji, Jian Bing, Bing Li, Lizhong Han, Haiqing Chu, Guanghua Huang

**Affiliations:** a Department of Respiratory and Critical Care Medicine, Shanghai Pulmonary Hospital, School of Medicine, Tongji University, Shanghai, China; b Department of Laboratory Medicine, Ruijin Hospital, Shanghai Jiao Tong University School of Medicine, Shanghai, China; c Shanghai Key Laboratory of Tuberculosis, Shanghai Pulmonary Hospital, School of Medicine, Tongji University, Shanghai, China; d Department of Clinical Microbiology, Ruijin Hospital, Shanghai Jiao Tong University School of Medicine, Shanghai, China; e Shanghai Institute of Infectious Disease and Biosecurity, Department of Infectious Diseases, Huashan Hospital and State Key Laboratory of Genetic Engineering, School of Life Sciences, Fudan University, Shanghai, China

**Keywords:** Fungemia, antifungal resistance, pan-echinocandin resistance, *Sporopachydermia lactativora*, emerging fungal pathogen

## Abstract

The cactophilic yeasts, *Sporopachydermia* species, are intrinsic resistance to echinocandins. We report the first case of fungemia caused by *S. lactativora* in China. *Sporopachydermia lactativora* could colonise and infect multiple animal tissues and could represent a new emerging fungal pathogen of humans and should not be ignored in clinical settings.

## 1. Introduction

*Sporopachydermia* species are members of the family Dipodascaceae and have traditionally been considered cactophilic yeasts (Moraes et al. 2005). However, recent studies have revealed their presence in diverse environmental niches, including buffalo faeces (Lorliam et al. 2013), filter membranes used in wine filtration (Perpetuini et al. 2021), and reverse osmosis membranes used in whey wastewater treatment (Vitzilaiou et al. 2020). Despite their environmental prevalence, human infections caused by *Sporopachydermia* species are uncommon, with only sporadic cases reported (Anoop et al. 2011; Kingston et al. 2017; Al Dallal et al. 2021). These findings suggest that yeasts such as *S. cereana* and *S. lactativora* could potentially emerge as opportunistic human pathogens.

To the best of our knowledge, only a single case caused by *S. lactativora* was reported in a drug abuser with a pulmonary infection in 2021 (Al Dallal et al. 2021). In this study, we describe the first case of fungemia caused by *S. lactativora* in a B-cell acute lymphoblastic leukaemia (B-ALL) in China. To explore its genetic and pathogenic features, we performed genomic and biological analyses and found that potential transmissions of *S. lactativora* strains between environmental and clinical settings could occur.

## 2. Case report

**Figure 1.**
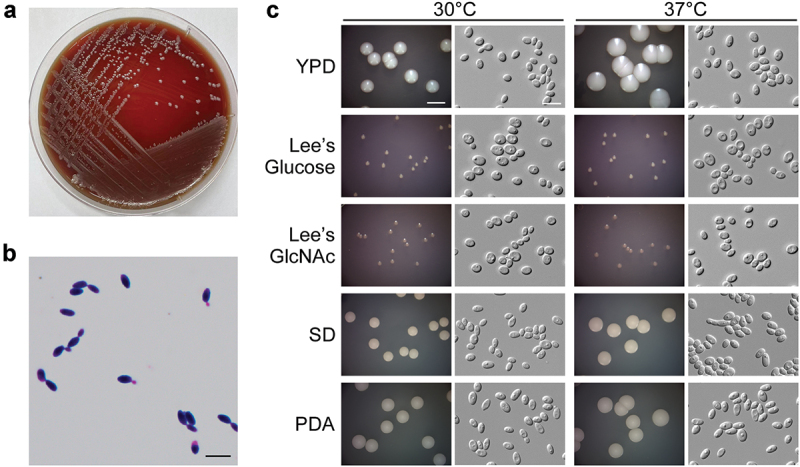
Morphologies of *Sporopachydermia lactativora* strain RJ001. (a) Colony morphology of *S. lactativora* grown on blood agar. The blood agar plate was incubated at 35 °C for 2 d. (b) Microscopic evaluation of Gram-stained *S. lactativora* cells. Scale bar, 10 µm. (c) Colony and cellular morphologies of *S. lactativora* strain RJ001 on YPD, Lee’s glucose, Lee’s GlcNAc (Xie et al. 2013), SD, and PDA media. Yeast cells were plated on the media and cultured at 30 °C or 37 °C for 5 d. Scale bar for colonies, 5 mm; for cells, 10 µm.

A 37-year-old female with pancytopenia and B-ALL was admitted to the hospital on 9 September 2023. Her white blood cell (WBC) count was 9.9 × 10^9^/L, C-reactive protein (CRP) level 45.50 mg/L, and procalcitonin level 0.12 ng/mL, respectively. Physical examination revealed abdominal distention and poor appetite. On the seventh day, the patient was treated with chemotherapy using the VDP regimen and subsequently developed severe myelosuppression and low fever. Blood culture became positive after 2 d of incubation at 35 °C, and white, smooth, and yeast-like colonies were found on the nutrient agar (Figure 1a), with fungal ellipsoidal cells observed by microscopic and Gram-staining assays (Figure 1b). Hyphae or pseudohyphae formation was not observed (Figures S1 and 1C), but elongated cells could occasionally be found under certain culture conditions (Figure 1c). The yeast cells grown on the agar plate were identified as *S. lactativora* by sequencing the ITS regions (GenBank accession number PQ289620). After three days of treatment with oral voriconazole (200 mg/tablet, two tablets daily), the blood culture became negative for fungi. The treatment regimen for B-ALL was continued. The patient improved with antifungal and B-ALL treatments and was discharged from the hospital on the 38^th^ day.

The antifungal susceptibility of *S. lactativora* to nine antifungal drugs (fluconazole, posaconazole, itraconazole, voriconazole, amphotericin B, caspofungin, anidulafungin, micafungin, and 5-fluorocytosine) was tested following the NCCLS document M27 method (Table 1). Briefly, cells of each strain were initially plated on YPD solid medium at 30 °C for 2 d and then washed with ddH_2_O. Approximately 500 cells were suspended in 200 µL liquid RPMI 1640 medium and incubated in 96-well U-bottom microplates at 35 °C for 24 h. Three biological repeats were performed. *Candida krusei* ATCC 6258 and *C. parapsilosis* ATCC 22019 were used as quality control strains. The results showed that *S. lactativora* strain RJ001 had high values of minimal inhibitory concentrations (MICs) for echinocandin drugs (16.0 µg/mL for caspofungin, CAS; 4.0 µg/mL for anidulafungin, AFG; and 4.0 µg/mL for micafungin, MFG). However, this strain was relatively susceptible to azoles, amphotericin B (AmB), and 5-fluorocytosine (5-FC). A striking feature of *Sporopachydermia* species is pan-echinocandin resistance. The thick cell wall of *S. lactativora* cells could contribute to the increased resistance to echinocandins (Kreger-van Rij 1978), which target the glucan synthase enzyme.

**Table 1.** Antifungal susceptibility testing of *Sporopachydermia lactativora* RJ001.

	FLC	POC	ITC	VOC	AmB	CAS	AFG	MFG	5-FC
MIC (µg/mL)	4	0.5	0.5	0.03125	1	16	4	4	0.02

FLC, fluconazole; POC, posaconazole; ITC, itraconazole; VOC, voriconazole; AmB, amphotericin B; CAS, caspofungin; AFG, anidulafungin; MFG, micafungin; 5-FC, 5-fluorocytosine; MIC, minimal inhibitory concentration.

*Sporopachydermia lactativora* and its closely related species *S. cereana* belong to the *Sporopachydermia* genus of the family *Dipodascaceae*. Based on the genomic sequences of *S. lactativora* and several related fungal species, we established a phylogenetic tree (Figure S2). Using the publicly available 18S rDNA sequences of *S. lactativora* strains and the strain isolated in this study (RJ001), we performed intra-species phylogenetic analysis and found two genetic clades (Figure S3). Both genetic clades include *S. lactativora* strains from environmental ecological niches and clinical settings, indicating that potential transmission between environmental and clinical settings occurred.

**Figure 2.**
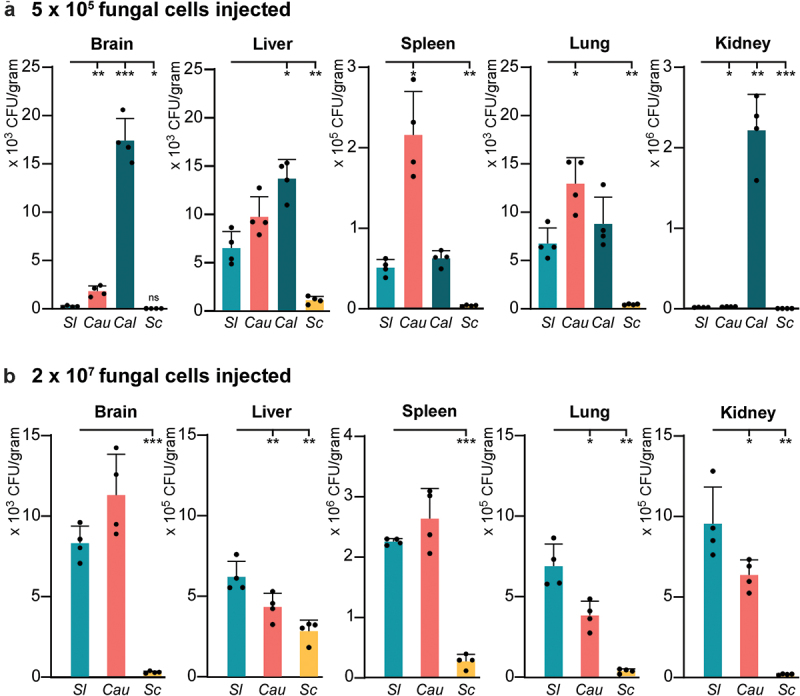
Fungal burden analyses of *Sporopachydermia lactativora* (*Sl*), *Candida auris* (*Cau*), *Candida albicans* (*Cal*), and *Saccharomyces cerevisiae* (*Sc*) in a mouse systemic infection model. Strains used: *Sporopachydermia lactativora* (RJ001), *C. auris* [BJCA001(Wang et al. 2018)], *C. albicans* (SC5314), and *Saccharomyces cerevisiae* (∑10560-2D). Four 6-week-old female BALB/c mice were used for systemic infection with each fungal strain. Cells of each strain were initially plated on YPD solid medium at 30 °C for 2 d, washed and then diluted with 1 × PBS. For each mouse, 5 × 10^5^ (panel A) or 2 × 10^7^ fungal cells (panel B) were injected via the tail vein. After 24 h of infection, the mice were euthanised, and five organs were collected, weighed, ground, and plated on YPD solid medium supplemented with chloramphenicol (final concentration, 34 µg/mL) for fungal burden assays. The statistical significance of the difference between *Sporopachydermia lactativora* and other species is indicated (*, *p* < 0.05; **, *p* < 0.01; ***, *p* < 0.001; two tailed student’s *t-*test). Black dots represent the CFU. Error bars represent standard deviations. Of note, when 2 × 10^7^ *C. albicans* cells were injected into the mice, all mice died within 24 h. Therefore, fungal burden data for *C. albicans* at this inoculation were not obtained.

To evaluate the pathogenesis of *S. lactativora*, we next performed fungal burden assays in a mouse systemic infection model and compared them to those of *C. albicans*, *C. auris*, and *Saccharomyces cerevisiae*. Four 6-week-old female BALB/c mice were used for systemic infection with each fungal strain. Cells of each strain were initially plated on YPD solid medium at 30 °C for 2 d, washed and then diluted with 1 × PBS. Fungal cells (5 × 10^5^ or 2 × 10^7^ cells per mouse) were injected via the lateral tail vein. After 24 h of infection, the mice were euthanised. As shown in Figure 2a, *S. lactativora* strain RJ001 exhibited a lower fungal burden than that of *C. albicans* and *C. auris* in all five organs (brain, liver, spleen, lung, and kidney) tested when injected with 5 × 10^5^ cells. However, the fungal burden of the *S. lactativora* strain was comparable to that of *C. auris* and much higher than that of *Saccharomyces cerevisiae* when injected with 2 × 10^7^ cells (Figure 2b). These results suggest that the infection ability of *S. lactativora* could be dose-dependent.

## 3. Discussion

The occurrence of new human fungal pathogens is on the rise, perhaps due to the wide use of a few classes of antifungal drugs in clinical settings and agriculture. Multidrug-resistant fungal pathogens are becoming a growing threat to public health (Ratemo and Denning 2023). In this study, we report the first case of blood fungal infection caused by a rare yeast *S. lactativora* in a general hospital in China.

The genus *Sporopachydermia* was named in 1978 because of the extraordinarily thick spore wall (Kreger-van Rij 1978; Rodrigues de Miranda 1978). Three ascomycetous yeasts, *Sporopachydermia lactativora*, *S. cereana*, and *S. quercuum* have been identified in this genus. Environmental *S. lactativora* strains were often isolated from Antarctic sea-water or waste lagoon, while *S. cereana* strains were mostly found in rotten stems of cactus species (Rodrigues de Miranda 1978; Anoop et al. 2011). In contrast with other pathogenic fungi, *Sporopachydermia* species were seldom reported in clinical settings. Only five cases of *S. cereana* and one case of *S. lactativora* infection have been reported around the globe (Al Dallal et al. 2021).

The *S. lactativora* strain isolated in this study exhibits intrinsic resistance to echinocandins, especially to caspofungin. Consisting with previous MIC assays of *Sporopachydermia* species (Al Dallal et al. 2021), echinocandin antifungals are not effective antimicrobial agents in the treatment of *S. lactativora* infections. Here, we added this echinocandin-resistant fungus to the list of emerging fungal pathogens. Our study of pathogenicity indicates that *S. lactativora* can colonise and infect multiple animal tissues and exhibits comparable virulence to the emerging fungal pathogen *C. auris* under certain conditions.

In summary, *S. lactativora* may represent a new opportunistic pathogen to humans. Given its resistance to echinocandins and tissue colonisation ability, this yeast and its phylogenetically related *Sporopachydermia* species should not be ignored in clinical settings.

## Supplementary Material

Supplemental Material
