# Supplementary material for: First case of fungemia caused by a rare and pan-echinocandin resistant yeast Sporopachydermia lactativora in China
Source: Mycology. 2024 Oct 28;16(2):956–60. doi: 10.1080/21501203.2024.2418111 (PMC12096653; doi:10.1080/21501203.2024.2418111)
Supplement: Supplemental Material [file TMYC_A_2418111_SM4052.docx]

Supplementary data for

**First case of fungemia caused by a rare and pan-echinocandin resistant yeast** ***Sporopachydermia lactativora* in China**

Qiushi Zheng^a#^, Shuzhen Xiao^b#^, Lingyu Ji^b#^, Jian Bing^b#^, Bing Li^a,c*^, Lizhong Han^b,d*^, Haiqing Chu^a,c*^, and Guanghua Huang^a,c,e*^

**
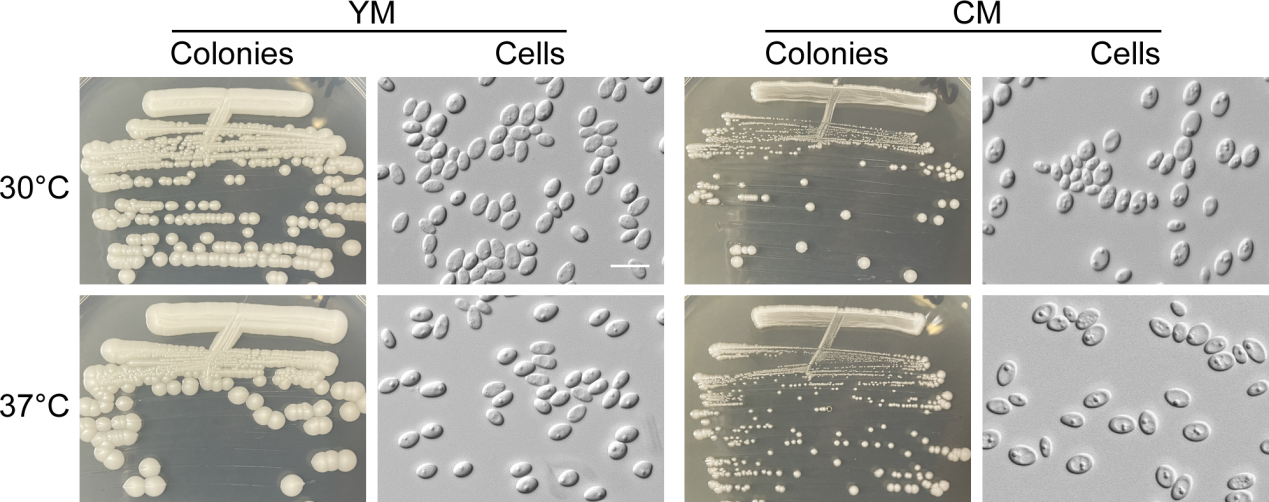
**

**Figure S1. Colony and cellular morphologies of *Sporopachydermia lactativora* strain RJ001 on YM and CM media.** Yeast cells were streaked on the media and cultured at 30 °C or 37 °C for 5 d. Scale bar for cells, 10 µm.


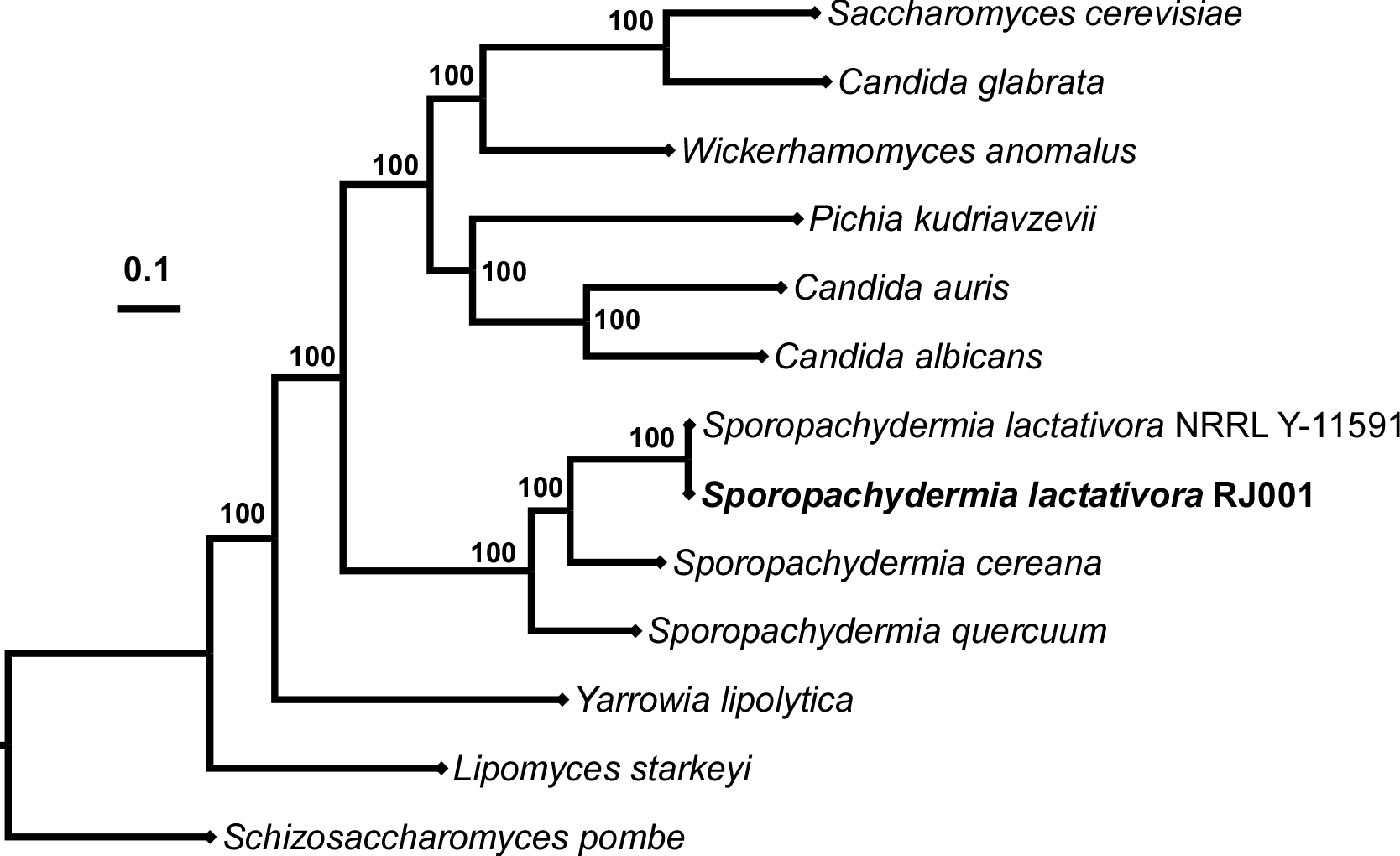


**Figure S2. Phylogenetic analysis of *Sporopachydermia lactativora* and associated fungal species based on the Maximum-Likelihood (ML) method.** A total of 1,119 orthologous proteins of *S. lactativora* strain RJ001 and other fungal species were analyzed using Mafft v7.402. The ML phylogenetic tree was generated using RAxML v7.3.2 with the JTT matrix-based model and 1,000 bootstrap replicates. The scale bar indicates nucleotide substitutions per site. *Schizosaccharomyces pombe* was used as an outgroup.

**
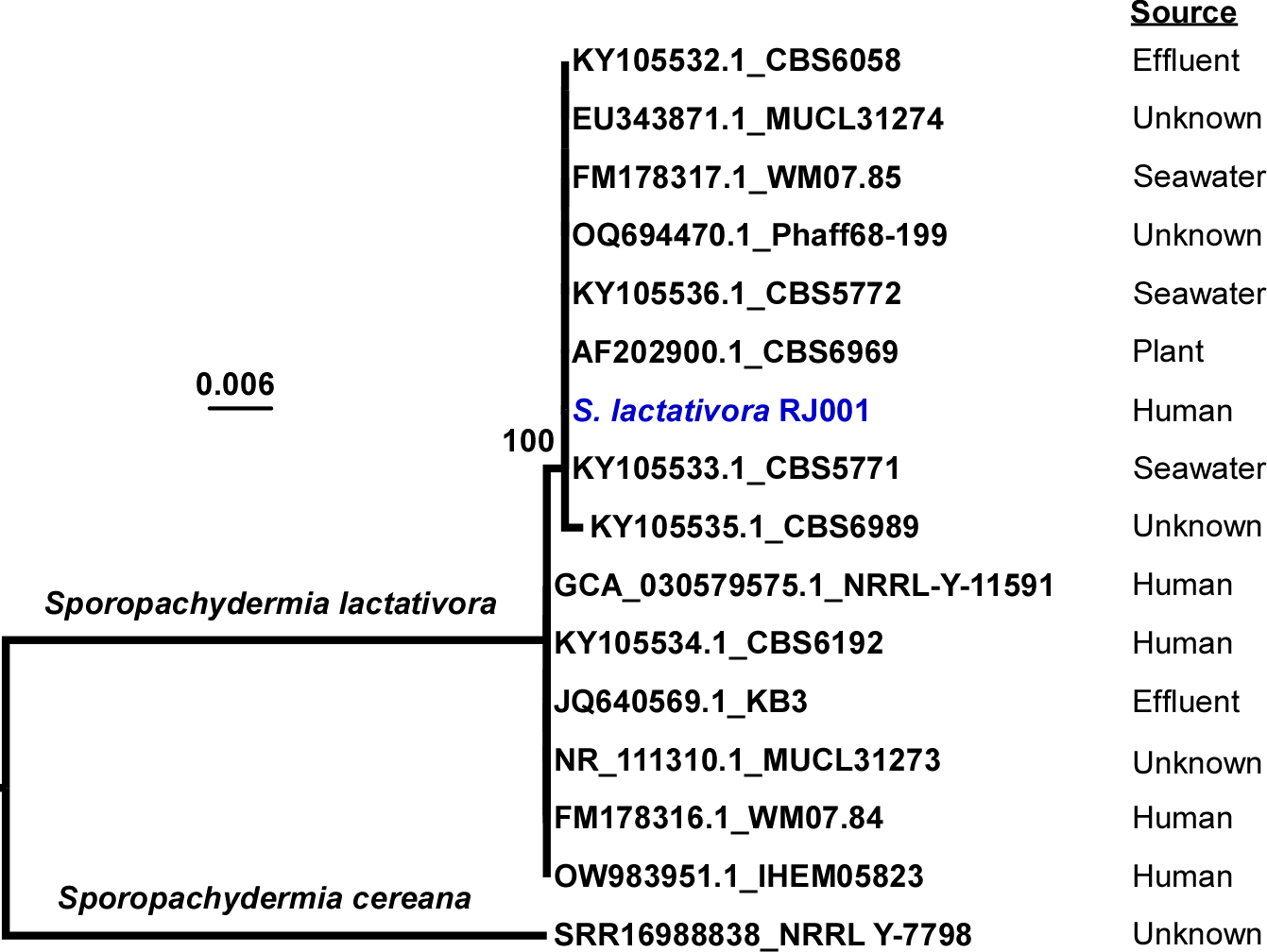
**

**Figure S3. Phylogenetic tree of *Sporopachydermia lactativora* strains from different sources.** The internal transcribed spacer (ITS) sequences of *S. lactativora* isolates were used to generate the ML phylogenetic tree using RAxML based on the General Time Reversible (GTR) model, Gamma distribution with Invariant sites (G + I), and 1,000 bootstrap replicates. The NCBI access number and strain name are shown for each isolate (e.g., KY105532.1_CBS6058, KY105532.1 represents the access number, and CBS6058 is the strain name). The scale bar indicates nucleotide substitutions per site. *S**poropachydermia cereana* (a species closely related to *S. lactativora*) was used as an outgroup. *Sporopachydermia lactativora* strain RJ001 was highlighted in blue. Ecological source of each strain is shown to the right of the phylogenetic tree.

**Materials and methods**

**1. Strains and culture**

Strains of *Sporopachydermia lactativora* (RJ001), *C. auris* (BJCA001), *C. albicans* (SC5314), and *S. cerevisiae* (∑10560-2D) were used in this study. Fungal strains were routinely grown on solid YPD medium (2% Glucose, 2% peptone, 1% yeast extract, 2% agar). Cornmeal (CM), yeast extract-malt extract (YM; 1% Glucose, 0.5% peptone, 0.3% yeast extract, 0.3% malt extract, pH 5.0), Lee’s glucose, Lee’s GlcNAc (Xie et al. 2013), SD, and PDA media were used for morphological analysis. Approximately 100 fungal cells were plated on each medium plate and incubated at 25°C, 30°C, or 37°C for 5 d.

**2. Antifungal susceptibility testing**

Antifungal susceptibility testing assays were conducted following the NCCLS document M27 method. Nine antifungal drugs (fluconazole, posaconazole, itraconazole, voriconazole, amphotericin B, caspofungin, anidulafungin, micafungin, and 5-fluorocytosine) were tested. *Candida krusei* ATCC 6258 and *Candida parapsilosis* ATCC 22019 were used as quality control strains. Cells of each strain were initially plated on YPD solid medium at 30°C for 2 d and then washed with ddH_2_O. Approximately 500 cells were suspended in 200 µL liquid RPMI 1640 medium and incubated in 96-well U-bottom microplates at 35°C for 24 h. Three biological repeats were performed.

**3. Genomic and phylogenetic analysis**

Yeast cells were grown on YPD medium at 30°C for 24 h. Genomic DNA was extracted using the TIANamp Yeast DNA Kit (TianGen Biotech, Beijing, China) following the recommended protocols. Short-insert fragment libraries were constructed according to the manufacturer’s protocol. Whole genome sequencing (WGS) was performed using the PE150 sequencing and DNBseq tech platform (BGISEQ), generating at least 3 GB of clean data per sample.

For species phylogenetic analysis, sequences of 1,119 orthologous proteins from 12 fungal species were aligned using Mafft v7.402 (Byrne and Wolfe 2005; Katoh and Standley 2013). The maximum likelihood phylogenetic tree was generated using the program RAxML v7.3.2 with the JTT matrix-based model. ITS sequences were used to construct the phylogenetic tree of *S. lactativora* isolates from different ecological sources. The tree was constructed using RAxML based on the General Time Reversible (GTR) model, Gamma distribution with Invariant sites (G + I), and 1,000 bootstrap replicates.

**4. Fungal burden assays**

All animal experiments were conducted following the guidelines approved by the Animal Care and Use Committee of Fudan University. Four 6-week-old female BALB/c mice were used for systemic infection with each fungal strain. Cells of each strain were initially plated on YPD solid medium at 30°C for 2 d, washed and then diluted with 1 x PBS. Fungal cells (5 x 10^5^ or 2 × 10^7^ cells per mouse) were injected via the lateral tail vein. After 24 h of infection, the mice were euthanized. The brain, liver, spleen, lung, and kidney tissues were collected, weighed, ground, and plated on YPD solid medium supplemented with chloramphenicol (final concentration, 34 µg/mL) for CFU assays.

**References**

Byrne KP, Wolfe KH. 2005. The yeast gene order browser: combining curated homology and syntenic context reveals gene fate in polyploid species. Genome Res. 15(10):1456–1461. doi: 10.1101/gr.3672305.

Katoh K, Standley DM. 2013. MAFFT multiple sequence alignment software version 7: improvements in performance and usability. Mol Biol Evol. 30(4):772–780. doi: 10.1093/molbev/mst010.

Xie J, Tao L, Nobile CJ, Tong Y, Guan G, Sun Y, Cao C, Hernday AD, Johnson AD, Zhang L, et al. 2013. White-opaque switching in natural MTLa/α isolates of *Candida albicans*: evolutionary implications for roles in host adaptation, pathogenesis, and sex. PLoS Biol. 11(3):e1001525. doi: 10.1371/journal.pbio.1001525.
